# Supplementary material for: Controlled Release of the α-Tocopherol-Derived Metabolite α-13′-Carboxychromanol from Bacterial Nanocellulose Wound Cover Improves Wound Healing
Source: Nanomaterials (Basel). 2021 Jul 28;11(8):1939. doi: 10.3390/nano11081939 (PMC8398652; doi:10.3390/nano11081939)
Supplement: Supplementary file 1 [file nanomaterials-11-01939-s001.zip › nanomaterials-1287520-supplementary.pdf]

nanomaterials

MDPI

supplementary

Controlled Release of the α-Tocopherol-Derived Metabolite α-13′-Carboxychromanol from Bacterial Nanocellulose Wound Cover Improves Wound Healing

Jessica Hoff [1,2], Berit Karl [3], Jana Gerstmeier [4], Uwe Beekmann [3], Lisa Schmölz [5,6], Friedemann Börner [4], Dana Kralisch [3,7], Michael Bauer [1,2,7], Oliver Werz [4,7], Dagmar Fischer [3,7,8], Stefan Lorkowski [5,6,7,*] and Adrian T. Press [1,2,9,*]

1 Department of Anesthesiology and Intensive Care Medicine, Jena University Hospital, Am Klinikum 1, 07747 Jena, Germany; jessica.hoff@med.uni-jena.de (J.H.); michael.bauer@med.uni-jena.de (M.B.)
2 Center for Sepsis Control and Care (CSCC), Jena University Hospital, Am Klinikum 1, 07747 Jena, Germany
3 Pharmaceutical Technology and Biopharmacy, Institute of Pharmacy, Friedrich Schiller University Jena, Lessingstraße 8, 07743 Jena, Germany; berit.karl@uni-jena.de (B.K.); beekmann@jenacell.de (U.B.); dana.kralisch@uni-jena.de (D.K.); dagmar.fischer@fau.de (D.F.)
4 Department of Pharmaceutical and Medicinal Chemistry, Institute of Pharmacy, Friedrich Schiller University Jena, Philosophenweg 14, 07743 Jena, Germany; jana.gerstmeier@uni-jena.de (J.G.); friedemann.boerner@uni-jena.de (F.B.); oliver.werz@uni-jena.de (O.W.)
5 Institut of Nutritional Sciences, Friedrich Schiller University Jena, Dornburger Straße 25, 07743 Jena, Germany; lisa.schmoelz@uni-jena.de
6 Competence Cluster for Nutrition and Cardiovascular Health (nutriCARD) Halle-Jena-Leipzig, Dornburger Straße 25, 07743 Jena, Germany
7 Jena Center for Soft Matter (JCSM), Friedrich Schiller University Jena, Philosophenweg 7, 07743 Jena, Germany
8 Department of Chemistry and Pharmacy, Divison of Pharmaceutical Technology, Friedrich Alexander University Erlangen-Nürnberg, Cauerstrasse 4, 91058 Erlangen, Germany
9 Medical Faculty, Friedrich Schiller University Jena, Bachstr. 18, 07743 Jena, Germany
* Correspondence: stefan.lorkowski@uni-jena.de (S.L.); adrian.press@med.uni-jena.de (A.T.P.)

Supplementary Figures

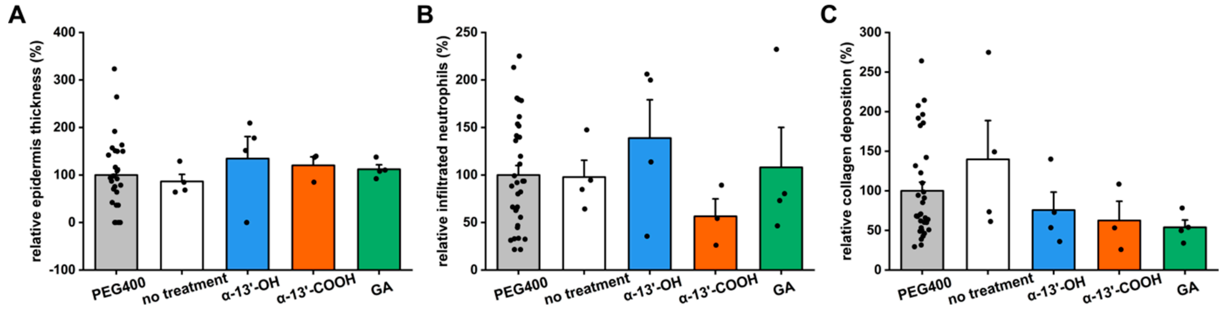

Figure S1. Histological analysis of wounds treated with low doses of tocopherol derivatives. (A) Relative epidermis thickness as measured and compared to vehicle-treated wounds. (B) Infiltrated neutrophils after 10 days. (C) Collagen deposition in the newly generated tissue was analyzed by second harmonic generation imaging as a measure of potential scar formation. Data are represented as mean bar plots with S.E. and individual data points. *unpaired t-test compared to PEG400 control, $p < 0.05$.

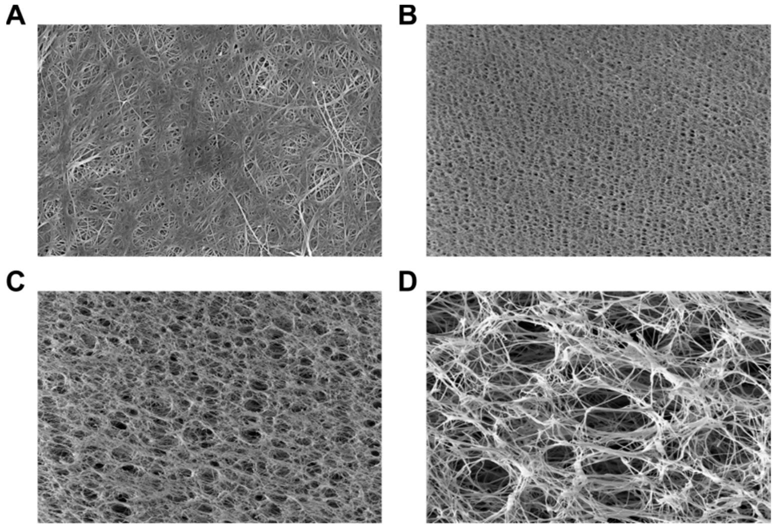

Figure S2. Representative pictures of the morphological structure of the native BNC network. Pictures are taken by scanning electron microscopy in different magnifications. Cross-sections of freeze-dried BNC samples were sputter-coated with gold before imaging. (A) Dense top layer. (B)-(D) Porous middle layer. Magnification: (A) 5000x, (B) 300x, (C) 100x and (D) 5000x.

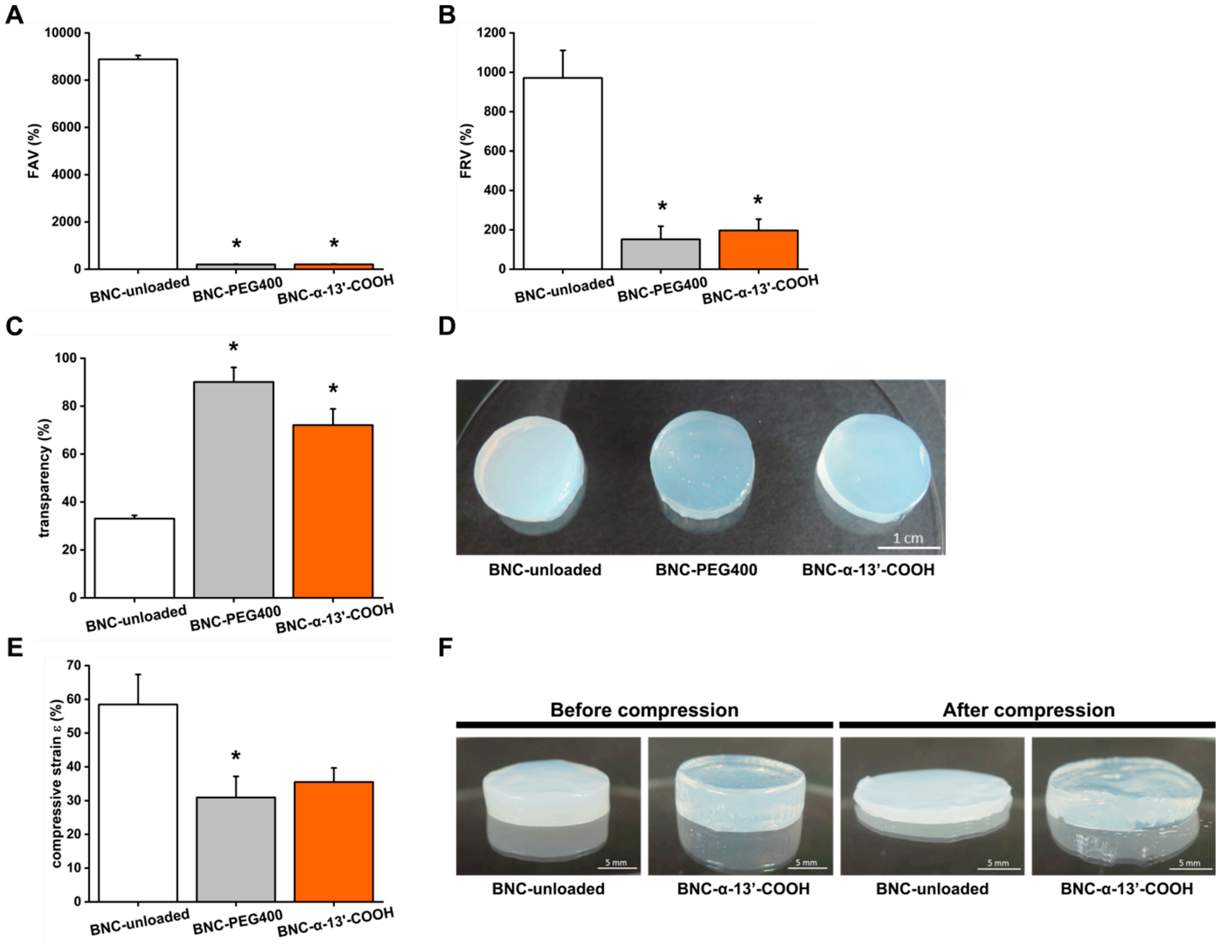

Figure S3. Physicochemical characterization of the bacterial nanocellulose (BNC). (A) Fluid absorption value (FAV) calculated by the weight of BNC determined before and after drying at 50 °C until constant mass. Comparison of the FAV of unloaded BNC, BNC loaded with PEG400 (BNC-PEG400), and BNC loaded with α-13′-hydroxychromanol (α-13′-COOH) dissolved in PEG400 (BNC-α-13′-COOH) (n = 2 in triplicates). (B) Fluid retention value (F.R.V.) calculated by the weight of BNC determined before and after centrifugation at 1073 g for 15 min and drying at 50 °C until constancy of mass of unloaded BNC compared to BNC loaded with PEG400 and BNC loaded with α-13′-COOH (n = 2 in triplicates). (C) Absorption measurement at 600 nm to show BNC transparency after incorporating PEG400 and α-13′-COOH dissolved in

PEG400 compared to unloaded BNC (n = 2 in quintuplicates). (D) Plan views of BNC samples visualizing increased transparency. (E) Compressive strain (ε) of unloaded BNC samples in comparison to samples loaded for 48 h with PEG400 or a solution of α-13′-COOH dissolved in PEG400. Each fleece was burdened with a weight of 400 g for 10 min (n = 2 in quadruplicates). (F) Compression stability of BNC samples loaded with 0.16 µg mL^{-1} α-13′-COOH dissolved in PEG400 in comparison to unloaded BNC (BNC-unloaded) sample. Both fleeces were compressed with 400 g for 10 min. Data are represented as mean bar plots with S.E. and individual data points. *unpaired t-test compared to unloaded control, p < 0.05.

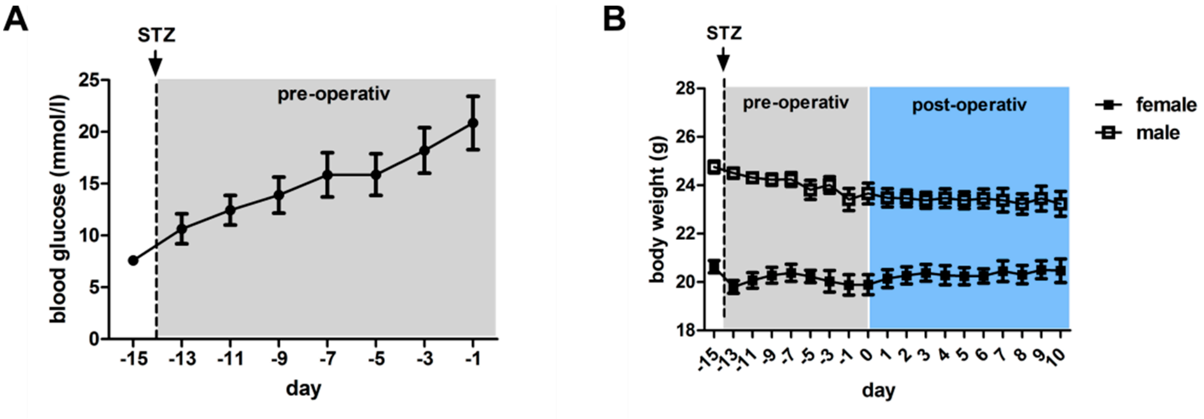

Figure S4. Analysis of blood glucose concentration and body weight after streptozotocin (STZ) injection. (A) Blood glucose and (B) body weight development pre-and post-operative. Wound healing was assessed in the splinted wound model over 10 days. n = 16, mean ± S.E.

Supplementary Tables

Table S1. Weight and blood glucose concentrations. (n = 16, mean ± S.E.).

day	0	2	4	6	8	10	12	14
**body weight (g)**	23.2 ± 2.2	22.7 ± 2.4	22.7 ± 2.2	22.7 ± 2.1	22.7 ± 2.1	22.3 ± 2.0	22.4 ± 2.2	22.0 ± 2.1
**blood glucose (mmol L^{-1})**	7.6 ± 0.8	10.6 ± 5.8	12.4 ± 5.7	13.9 ± 7.0	15.9 ± 8.5	15.9 ± 8.1	18.2 ± 8.8	20.9 ± 10.3

Table S2. Used RT-qPCR primers. In each case, forward and reverse primers are located in different exons.

gene symbol	gene name	GenBank	forward primer (from 5' to 3')	reverse primer (from 5' to 3')	product size [bp]
**COL1A1**	Collagen 1A1	NM_000088	TACAGCGTCAC-TGTCGATGGC	TCAATCACTGTCTT-GCCCCAG	61
**MMP2**	Matrix metalloprotein-ase-2	NM_004530<br>NM_001127891	ATTTGATGG-CATCGCTCAGATC	TCACGTGGCGTCACAGTCC	81
**TIMP1**	tissue inhibitor of met-alloproteinase 1	NM_003254	GATCCAGCGCCCAGAGA-GAC	GCTATCAGCCACAG-CAACAACAG	91
**TIMP2**	tissue inhibitor of met-alloproteinase 2	NM_003255	GAGTTTATCTACAC-GGCCCCCTC	CACTCG-CAGCCCATCTGGTA	203
**TIMP3**	tissue inhibitor of met-alloproteinase 3	NM_000362	TCGGCACGCTGGTCTACAC	GCTGGTCCCACCTCTCCAC	210

Supplementary Methods

Scanning electron microscopy

For evaluation and characterization of the BNC network structure, BNC samples were freeze-dried by lyophilization at −80 °C and 0.01 mbar for 72 h using the freeze dryer Alpha 2–4 L.D. plus (Martin Christ Gefriertrocknungsanlagen, Osterode am Harz, Germany). Hereinafter, pictures of BNC cross-sections were taken by scanning electron microscopy with the help of a Leica S440i scanning electron microscope (Leica Microsystems, Wetzlar, Germany).

Fluid binding capacity

Fluid absorption value (*FAV*) and *fluid retention value* (*FRV*) were examined for unloaded (BNC-unloaded), BNC-PEG400, and drug (α-13′-COOH dissolved in PEG400) loaded BNC (BNC-COOH) samples. Fleeces were cut into four equally sized pieces and re-swollen in loading solution for 2 h. Weight (*Wi*) and dimension were determined by drying at 50 °C in a drying cabinet (W.T.C. Binder, Tuttlingen, Germany) until constancy of mass. Samples were weighed again (*Wd*), and *FAV* was calculated with the following equation:

$$FAV = Wi/Wd * 100\%$$

Data for *FRV* were received by slicing two equally sized pieces of the BNC fleeces followed by incubation for 2 h in loading solution. After centrifugation at 1073 g for 15 min, drying was performed in a drying cabinet (W.T.C. Binder, Tuttlingen, Germany) at 50 °C until constancy of mass. The weight and dimensions of the BNC pieces were determined before and after drying. *FRV* was calculated according to the equation:

$$FRV = (Wi - Wd/Wd) * 100\%$$

Wi represents the weight of the dry BNC fleece, and *Wd* represents the weight after centrifugation.

Measurement of transparency

Samples with comparable heights were selected. They were measured in 24-well plates (Greiner bio-one, Frickenhausen, Germany) by spectrophotometry in the plate reader Tecan Spark 10 M (Tecan Austria, Grödig, Austria) at 600 nm [1].The mean absorption of the samples was shown relatively to maximum absorption.

Mechanical stability

BNC samples were characterized regarding their height and weight at room temperature, as described in previous sections. A weight of 400 g was placed on the BNC fleeces for 10 minutes. After removal, height and weight determination were performed again to calculate compressive strain ε and weight reduction. The following equation evaluated the compressive strain ε:

$$\varepsilon = \Delta h/h_0 * 100\%$$

Δh and the height of BNC show the change of height before compression by h_0 (mm). The calculation of weight reduction was conducted according to the formula:

$$m_{loss} = \Delta m/m_0 * 100\%$$

Δm represents mass changes due to compression, and m_0 represents mass before compression.

References

1. Gutiérrez, T.J.; Alvarez, V.A. Data on physicochemical properties of active films derived from plantain flour/PCL blends developed under reactive extrusion conditions. *Data Brief* **2017**, *15*, 445–448, doi:10.1016/j.dib.2017.09.071.
